# Percutaneous Lead Extraction in Infection of Cardiac Implantable Electronic Devices: a Systematic Review

**DOI:** 10.21470/1678-9741-2017-0144

**Published:** 2018

**Authors:** Antônio da Silva Menezes Júnior, Thaís Rodrigues Magalhães, Alana de Oliveira Alarcão Morais

**Affiliations:** 1 Escola de Ciências Médicas, Farmacêuticas e Biomédicas of the Pontifícia Universidade Católica de Goiás (PUC-GO), Goiânia, GO, Brazil.

**Keywords:** Infection, Review Literature as Topic, Review, Cardiac Resynchronization Therapy Devices, Device Removal/Methods

## Abstract

**Introduction:**

In the last two decades, the increased number of implants of cardiac implantable electronic devices has been accompanied by an increase in complications, especially infection. Current recommendations for the appropriate treatment of cardiac implantable electronic devices-related infections consist of prolonged antibiotic therapy associated with complete device extraction. The purpose of this study was to analyze the importance of percutaneous extraction in the treatment of these devices infections.

**Methods:**

A systematic review search was performed in the PubMed, BVS, Cochrane CENTRAL, CAPES, SciELO and ScienceDirect databases. A total of 1,717 studies were identified and subsequently selected according to the eligibility criteria defined by relevance tests by two authors working independently.

**Results:**

Sixteen studies, describing a total of 3,354 patients, were selected. Percutaneous extraction was performed in 3,081 patients. The average success rate for the complete percutaneous removal of infected devices was 92.4%. Regarding the procedure, the incidence of major complications was 2.9%, and the incidence of minor complications was 8.4%. The average in-hospital mortality of the patients was 5.4%, and the mortality related to the procedure ranged from 0.4 to 3.6%. The mean mortality was 20% after 6 months and 14% after a one-year follow-up.

**Conclusion:**

Percutaneous extraction is the main technique for the removal of infected cardiac implantable electronic devices, and it presents low rates of complications and mortality related to the procedure.

**Table:** 

Abbreviations, acronyms & symbols	
BVS	= Biblioteca Virtual em Saúde
CENTRAL	= Cochrane Register of Controlled Trials
CI	= Confidence Interval
CIED	= Cardiac Implantable Electronic Devices
DMP	= Data Management Platform
ICD	= Implantable Cardioverter-defibrillator
MeSH Terms	= Medical Subject Headings Terms
PRISMA	= Preferred Reporting Items for Systematic Reviews and Meta-Analyses
PubMed	= US National Library of Medicine
SciELO	= Scientific Electronic Library Online
VHL	= Virtual Health Library

## INTRODUCTION

In the last two decades, the number of cardiac implantable electronic devices (CIED) has increased. CIED complications, of which infection is among the most important, have also increased. In the United States, 2.9 million patients had a permanent pacemaker between 1993 and 2009, which represents an increase in pacemaker use of 55.6% during this period^[1]^. At the same time, there was a 210% increase in CIED infections, which is alarming because these infections represent a serious and costly complication for the health care system^[2]^. In a large population study, the incidence of CIED-related infection was estimated at 1.82 for each 1,000 implanted devices per year between 1982 and 2007^[3]^.

The appropriate treatment of CIED-related infections is the administration of prolonged antibiotic therapy associated with complete device extraction. The importance of removing the device was evidenced after an analysis of infected leads, which demonstrated that bacteria coated the leads and formed a "biofilm", making the infection resistant to antibiotics^[4]^. Thus, non-removal of the device is associated with an increased risk of infection recurrence and device-related endocarditis, in addition to increased patient mortality^[5]^.

There are two types of procedures for the removal of leads: surgical removal and percutaneous extraction. Surgical removal is performed by thoracotomy and extracorporeal circulation and presents a high mortality rate, ranging from 12.5% to 21%; it is mainly reserved for cases requiring repair of valve injury, large vegetations or failure after percutaneous attempt. When comparing aspects of the percutaneous removal and surgical removal, it is imperative to consider that the complications of surgical removal usually relate to the more severe patients selection, large vegetations, abscesses, including cases of septicemia, or even cases of complication in the attempt of percutaneous extraction. On the other hand, percutaneous extraction has been indiscriminately used in uncountable cases with uninfected leads in patients with an inferior profile concerning the procedure's risk^[6,7]^.

Infection related to intracardiac devices significantly increases morbidity and mortality rates as well as the costs for health services and the length of hospital stay^[8]^. Although evidence shows that adequate treatment of these infections consists of antibiotic therapy associated with CIED removal, preferably by percutaneous extraction, several treatment aspects remain uncertain in the literature. This review aims to analyze the importance of percutaneous lead extraction in the treatment of CIED infections.

## METHODS

A systematic review was conducted on lead extraction in the treatment of CIED-related infections. The review followed the guidelines defined by the UK Cochrane Center in an effort to reduce bias and provide reliable results^[9]^.

The search for the studies was conducted in the following databases: PubMed (US National Library of Medicine), Biblioteca Virtual em Saúde [BVS; Virtual Health Library (VHL)], Cochrane Central Register of Controlled Trials (CENTRAL), Portal de Periódicos CAPES (Portal of Journals CAPES), SciELO (Scientific Electronic Library Online) and ScienceDirect (Elsevier Science).

Regarding the search for articles, search filters specific to each database that were validated by the Cochrane Collaboration with a combination of terms using Boolean operators ("AND" and "OR") were used. Table 1 provides an overview of the search strategies and the number of identified articles according to the descriptors and terms defined in the different databases.

**Table 1:** Search strategy and results found in databases.

Databases	Terms used / Search strategy	Results
PubMed	((("pacemaker, artificial"[MeSH Terms] OR ("pacemaker"[All Fields] AND "artificial"[All Fields]) OR "artificial pacemaker"[All Fields] OR ("pacemaker"[All Fields] AND "artificial"[All Fields]) OR "pacemaker, artificial"[All Fields]) OR (("cardiovascular system"[MeSH Terms] OR ("cardiovascular"[All Fields] AND "system"[All Fields]) OR "cardiovascular system"[All Fields] OR "cardiovascular"[All Fields]) AND "implantable"[All Fields] AND ("electronics"[MeSH Terms] OR "electronics"[All Fields] OR "electronic"[All Fields]) AND ("equipment and supplies"[MeSH Terms] OR ("equipment"[All Fields] AND "supplies"[All Fields]) OR "equipment and supplies"[All Fields] OR "device"[All Fields]))) AND "infection/therapy"[Mesh Terms]) AND ((("lead"[MeSH Terms] OR "lead"[All Fields]) AND "extraction"[All Fields]) OR "extraction"[All Fields] OR ("transvenous"[All Fields] AND ("lead"[MeSH Terms] OR "lead"[All Fields]) AND "extraction"[All Fields]) OR ("device removal"[MeSH Terms] OR ("device"[All Fields] AND "removal"[All Fields]) OR "device removal"[All Fields]))	232
BVS	(tw:(marcapasso artificial cardíaco OR marca-passo artificial OR pacemaker, artificial)) AND (tw:(infecção OR infection)) AND (tw:(remoção de dispositivo OR device removal OR extraction)) AND (instance:"regional") (tw:(pacemaker, artificial OR cardiovascular implantable electronic device OR marcapasso cardíaco artificial OR marca-passo artificial)) AND (tw:(infection OR infecção)) AND (tw:(lead extraction OR extraction OR device removal OR remoção de dispositivo)) AND (instance:"regional")	229
CENTRAL	("cardiovascular implantable electronic device infection" OR "pacemaker infection") AND ("lead extraction" OR "lead removal" OR "device removal")	124
CAPES	("pacemaker artificial infection" OR "cardiovascular implantable electronic device infection") AND ("lead extraction" OR "device removal")	776
SciELO	(pacemaker infection) AND (lead extraction) OR (pacemaker infection treatment) (cardiovascular implantable electronic device) OR (pacemaker) AND (infection) AND (lead extraction)	21
ScienceDirect	(pacemaker infection OR cardiovascular implantable electronic device infection) AND (lead extraction OR transvenous lead extraction OR extraction OR treatment OR management)	335

The search was carried out in August 2016. Initially, the studies were screened by an exploratory reading of the title and abstract by the two researchers, acting independently. The criteria for initial inclusion of the studies were delimited by Relevance Test I, namely: (1) primary studies, except for case reports; (2) approach for percutaneous lead extraction in cardiac implantable electronic device infections; (3) age 18 years and over; (4) articles published in English or Portuguese; (5) articles published between 2009 and 2016, as 2009 was the year of publication of a consensus of the Heart Rhythm Society approved by the American Heart Association, which unified the opinions and disagreements regarding the indications for device extraction, highlighting infection as one of the three major categories^[10]^.

After the initial screening, duplicates of the articles were removed, and the complete text of each article was read. In this second phase, the articles were selected through Relevance Test II, in which the research problem, objectives, methodology and results of each study were analyzed in more detail to evaluate the quality of the selected study and to classify it as relevant or not to the review.

The studies were independently selected by two reviewers. Disagreements were resolved by consensus, and if this was not possible, they were resolved based on the decision of a third reviewer. Finally, the extracted data were interpreted and grouped in tables in order to facilitate comparative analysis of the articles and identification of the differences among them. The report of the systematic review was guided using the PRISMA (Preferred Reporting Items for Systematic Reviews and Meta-Analyses) checklist^[11]^.

Data were analyzed using RevMan 5.0 statistical software provided by Cochrane Collaboration. DMP and 95% CI were used as summary estimates. The presence of heterogeneity among the studies was tested with the χ^2^ heterogeneity test and the I^2^ statistic. Heterogeneity was significant when *P*<0.05 or I^2^ was greater than 50%. A random effects model was used in all analyzes to test the stability of the results at the choice of the statistical model. If there is significant heterogeneity, the results of the random effects model are used. A priori sensitivity analysis of high quality studies for each clinical outcome was performed. The potential for publication bias was evaluated using the funnel chart approach.

## RESULTS

**Fig. 1:**
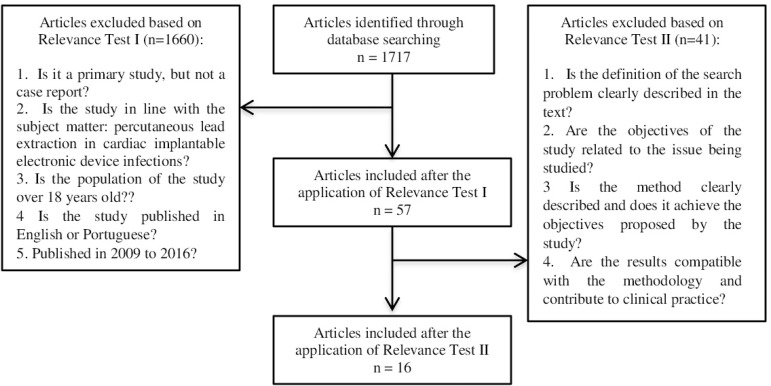
Flowchart of the systematic literature search in databases and of study selection.

Initially, 1,717 articles were identified by searching the research databases. Of these, after exclusion of the repeated studies that were indexed in more than one database and after the application of Relevance Test I, 57 articles were selected. Subsequently, Relevance Test II was applied, delimiting the final selection of 16 articles. In Figure 1, a diagram depicts the selections and the reasons for exclusion of the articles.

Following the literature search, 16 studies were included in this review, and their main characteristics are presented in Tables 2 and 3. Regarding the study method, 14 of the 16 studies performed retrospective analyses of the data recorded from patients with CIED-related infection in a given period of time. Only two references, Amraoui et al.^[12]^ and Deharo et al.^[13]^, consisted of prospective studies. In addition, the articles by Deharo et al.^[13]^, Rickard et al.^[14]^ and Cengiz et al.^[15]^ compared their results to a control group of patients with intracardiac devices but with no history of infection.

**Table 2:** Demographic characteristics and comorbidities of the patients and clinical presentation of the infection.

Characteristics of patients and clinical presentation	Number of patients
Age (years)	67.8 (58-73)
Comorbidities	
Coronary artery disease	888
Hypertension	802
Diabetes mellitus	537
Heart failure	590
Chronic renal failure	483
Atrial fibrillation	438
Chronic obstructive pulmonary disease	172
Immune suppression/corticosteroid	174
Malignancy	51
Use of anticoagulants	85
Signs / symptoms of local infection	
Purulent drainage	372
Erythema	347
Pain	270
Swelling	267
Warmth	197
Skin ulceration	113
Signs/symptoms of systemic infection	
Fever	628
Chills	280
Malaise	115
Signs of sepsis	127
Fatigue	27
Anorexia	20
Nausea	8
Endocarditis/vegetation	1029
Devices	
Pacemaker	1745
ICD	819
Biventricular	380

**Table 3:** Characteristics of selected studies in relation to device extraction and in-hospital and long-term mortality.

Author	Patients (number)	Method of extraction of intracardiac devices	Complications related to extraction (%)	Mortality during hospitalization (%)	Follow-up time (months)	Long-term mortality (%)
Greenspon et al.^16]^	129	Percutaneous: 112 Surgery: 17	Majors: 4.6 Minors: -	10.8	6	14.5
Rickard et al.^[14]^	151	Percutaneous: 151 Surgery:	-	6.6	24	-
Ipek et al. ^[20]^	34	Percutaneous: 28 Surgery: 5	Majors: 2.9 Minors: 14.7	8.8	-	-
Pichlmaier et al.^[25]^	178	Percutaneous: 144 Surgery: 34	Majors: 2.2 Minors: 14.0	3.9	Average of 55	18.5
Knigina et al.^[17]^	192	Percutaneous: 155 Surgery: 37	-	3.6	66	13.5
Grammes et al.^[21]^	100	Percutaneous: 100 Surgery: -	Majors: 2.0 Minors: 3.0	10.0	14.5	12.7
Tarakji et al.^[26]^	502	Percutaneous: 502 Surgery: -	-	5.0	12	20.3
Amraoui et al.^[12]^	100	Percutaneous: 100 Surgery: 2	Majors: 2.0 Minors: 6.0	2.0	12	4.0
Greenspon et al.^[18]^	145	Percutaneous: 145 Surgery: -	Majors: 4.8 Minors: -	6.2	6	27.6
Cengiz et al.^[15]^	57	Percutaneous: 17 Surgery: 18	-	3.5	-	-
Baman et al.^[19]^	210	Percutaneous: 170 Surgery: 17	Majors: 4.8 Minors:12.3	8.1	6	18.0
Goya et al.^[22]^	183	Percutaneous: 183 Surgery: 4	Majors: 2.7 Minors: 3.8	2.2	-	-
Deharo et al.^[13]^	197	Percutaneous: 189 Surgery: 13	Majors: 1.0 Minors: 12.2	4.1	Average of 25	1 year: 14.3 5 years: 35.4
Le et al.^[24]^	416	Percutaneous: 325 Surgery: 91	Majors: 4.1 Minors: 6.5	5.5	12	14.7
Gomes et al.^[23]^	348	Percutaneous: 348 Surgery: -	-	2.0	66	-
Tarakji et al.^[27]^	412	Percutaneous: 412 Surgery: -	Majors: 0.5 Minors: 3.4	4.6	6	17.0
Total	3354	Percutaneous: 3081 Surgery: 238	-	-	-	-
Mean	209.6	-	Majors: 2.9 Minors: 8.4	5.4	24	-

### Baseline Characteristics

The 16 studies included in this systematic review described a total of 3,354 patients diagnosed with CIED-related infection who underwent device removal. The duration of the selected studies ranged from two to 20 years, with a mean of 8.6 years. The mean age of the evaluated patients was 67.8 years. Table 2 presents the demographic and clinical characteristics of the patients analyzed in the studies.

In the articles by Greenspon et al.^[16]^, Knigina et al.^[17]^, Greenspon et al.^[18]^ and Baman et al.^[19]^, infection occurred after review or replacement of the intracardiac device in 306 out of 676 patients (45.3%). The mean time from the last procedure to the onset of infection was 29 months^[14,16,20-24]^. In Greenspon et al.^[18]^, the author divided the patients into two groups according to time of use of the device, considering a recent infection as one that occurred within 6 months of the most recent procedure in the device and a late infection as one that occurred after six months. Goya et al.^[22]^ defined a recent infection as one occurring within three months of the last procedure, a late infection as one occurring between four and 12 months, and a delayed infection as one that occurred after 12 months. In both studies, most patients had a later infection, namely, 71.8% and 85.3% of the patients in Greenspon et al.^[18]^. and Goya et al.^[22]^, respectively.

Regarding the types of infected CIED analyzed in the studies, there were 1,745 pacemakers, 819 implantable cardioverter defibrillators (ICD) and 380 biventricular devices with or without defibrillation function.

### Clinical Presentation

The articles selected for this study characterized the clinical presentation of patients through the signs and symptoms representative of local infection, systemic infection, and endocarditis or the identification of vegetations on the leads or heart valves. The results are shown in Table 2.

The articles by Pichlmaier et al.^[25]^, Tarakji et al.^[26]^, Amraoui et al.^[12]^, Baman et al.^[19]^, Goya et al.^[22]^ and Gomes et al.^[23]^ did not describe the signs and symptoms presented by the patients, classifying the infections only as local (1,152 patients) or systemic (562 patients).

The main signs and symptoms of local infection were local purulent drainage, erythema, pain, swelling, warmth and skin ulceration. The predominant manifestations of systemic infection were fever, chills, malaise, signs of sepsis, fatigue, anorexia and nausea.

The articles described 1,029 patients with endocarditis who were diagnosed by the modified Duke criteria or the presence of vegetation on echocardiography. The article by Greenspon et al.^[16]^ divided the presence of vegetation into two groups according to their size; the first group included patients with vegetation smaller than 1 cm, and the second group included patients with vegetation larger than 1 cm. Patients with smaller vegetation more frequently showed signs and symptoms of local infection, whereas the presentation of the systemic infection was more common in patients with larger vegetation.

In addition, the study by Greenspon et al.^[18]^ showed that signs of local infection were seen in most patients with recent infection (onset less than six months after the last device procedure), which is different from patients with late infection, who mostly presented signs of systemic infection.

### Device Extraction

All the selected articles addressed device removal as a treatment of CIED-related infections. Percutaneous or transvenous extraction was performed in 3,081 patients, and thoracotomy was performed in 238 cases, as shown in Table 3.

The main indications for surgical removal were the failure of transvenous extraction, large vegetations, vascular trauma in percutaneous extraction, the need for epicardial leads, concomitant valve involvement, abscesses, and tricuspid valve stenosis^[16,20,25]^.

In percutaneous extraction, the main technique consisted of simple manual traction of the cables, but some studies reported the need for more advanced techniques for proper removal of the device, such as laser sheath (504 patients), locking stylets (323 patients) and dilator sheaths (52 patients)^[14-17,22,24,25]^. The study by Gomes et al.^[23]^ demonstrated that patients with systemic infection more commonly required mechanical extraction equipment rather than simple traction.

The success rate for the complete removal of infected devices by percutaneous approach ranged from 83.3% to 97.6%, with a mean of 92.4%^[12,13,16,18,22,24,25]^.

### Complications

Complications related to lead extraction can be classified as major and minor^[10]^. Of the 16 evaluated studies, 11 articles reported the occurrence of complications related to the device extraction procedure in a total of 191 patients (60 majors and 131 minors). The incidence of major complications ranged from 0.5% to 4.8%, with a mean of 2.9%. On the other hand, the incidence of minor complications ranged from 3% to 14.7%, with an average of 8.4%.

The major complications presented in the studies were vascular or cardiac rupture (33.3%), pulmonary embolism (33.3%), cardiac tamponade (10%) and respiratory or anesthesia-related failure (6.7%). Regarding the minor complications, there was a predominance of pocket hematoma (31.3%), cable fragment migration or systemic embolization of vegetations without sequelae (30.5%), the need for blood transfusion (6.1%), and pericardial effusion without the need for pericardiocentesis (5.3%).

Recurrence of infection occurred in 52 patients. In Ipek et al.^[(20]^ study, conservative therapy with only antibiotics or failure to completely remove the infected device were considered predisposing factors for recurrence of infection.

In the study by Greenspon et al.^[16]^, the presence of larger vegetations was considered a risk factor for the occurrence of complications, and larger vegetations were also related to a greater frequency of changes in procedures for thoracotomy during the device removal attempt.

### Reimplantation

In the articles used for this systematic review, reimplantation of a new cardiac electronic device was considered in all patients with clinical indications. The new procedure was not performed when the patient died, in patients without a clinical indication or when the patient refused to receive a new device. In total, reimplants were reported in 1,402 patients. In most articles, the mean time between the removal of an infected device and the placement of a new device was within eight to 42 days^[14,20,21,25]^, except in Amraoui et al.^[12]^, in which reimplantation of a new epicardial pacemaker was performed during the same surgical procedure.

Rickard et al.^[14]^ observed that patients whose infected biventricular device was extracted and who were not subsequently reimplanted with a new device had worse results when compared to those patients who were reimplanted.

### In-Hospital Mortality

Mortality during the hospitalization of patients with CIED-related infection ranged from two to 10.8% in the studies, with an average of 5.4%. The main identified causes of in-hospital death were sepsis, multiorgan system failure, severe ventricular dysfunction, stroke, cardiorespiratory arrest, renal failure, septic shock and acute respiratory failure.

Greenspon et al.^[16]^ found no statistically significant correlation between in-hospital mortality and the size of vegetation presented by patients with endocarditis associated with intracardiac devices.

In addition, Knigina et al.^[17]^ also found no difference in mortality among the group of patients with recurrent infection compared to the group of patients with primary infection, i.e., no previous history of infected CIED.

Finally, Le et al.^[24]^ observed that patients with complications after device extraction were four times more likely to die when compared to those with a successful procedure.

In this review, the mortality directly related to the CIED extraction procedure ranged from 0.4% to 3.6%^[12,17,18,21,25-27]^.

### Follow-Up and Long-Term Mortality

In all the surveyed articles that reported follow-up of patients after hospital admission, the minimum observation time was six months. The mean follow-up time reported in the studies was 24 months. Table 3 presents the main characteristics regarding in-hospital and long-term mortality of the selected studies.

After six months of follow-up of the patients, some studies observed an average mortality of 20%^[16,18,19]^. Baman et al.^[19]^ demonstrated the following independent factors as predictors of mortality in this period: systemic embolization, right heart failure, moderate or severe tricuspid regurgitation and abnormal renal function. The size of the vegetation was not associated with a worsening of survival in six months^[16,19]^.

Regarding mortality after a one-year follow-up, the mean was 14%^[(12 13,24,26,27)]^. In Tarakji et al.^[26]^ study, the following were listed as risk factors for mortality within one year after treatment of CIED-related infections: dementia, chronic renal disease, advanced heart failure, the use of an anticoagulant, bleeding requiring blood transfusion, simultaneous infection and systemic infection. The presence of vegetation on echocardiography was not considered an important risk factor in relation to long-term mortality^[26]^.

In that same study, it was estimated that the presence of systemic infection was associated with an approximately twice as likely chance of death as the initial presentation of local infection^[26]^. In Deharo et al.^[(13]^ study, the one-year mortality rate did not present a statistically significant difference between the groups with local infection and endocarditis (12.5% and 15.5%, respectively).

In Le et al.^[(24]^ study, factors such as advanced age, greater number of comorbidities, longer time of cardiac implantation and use of corticosteroids or immunosuppressive therapy were considered to influence mortality. The author also showed that patients who did not have their devices removed (because of a high risk of complications or low life expectancy) presented a higher one-year mortality rate when compared to patients who had their devices removed. In addition, in this follow-up period, a three-fold increase in mortality was observed when the device extraction was delayed^[24]^.

In Rickard et al.^[14]^ paper, it was demonstrated that two years after the extraction of infected biventricular devices, the survival of patients who underwent subsequent reimplantation of a new cardiac device was similar to those who never contracted an implantable device infection. Le et al.^[24]^ also showed lower mortality in the one-year follow-up period for reimplanted patients compared to those patients who did not obtain a new device.

In the article by Knigina et al.^[17]^, the mean follow-up was 5.5 years (minimum of 2 years) and the identified mortality was 13.5%. The causes of death were not related to infection in 92.3% of the cases, and in the remaining patients (7.7%), septicemia was identified as the cause.

Deharo et al.^[13]^ demonstrated that mortality was 14.3% in one year and 35.4% in 5 years, but no statistically significant difference in mortality was found compared to a control group with non-infected CIED. In this study, advanced age, infected resynchronization device, thrombocytopenia (platelet count less than 100 Giga/l on admission), renal dysfunction and reimplantation of an epicardial pacemaker in the right ventricle were predictors of long-term mortality^[13]^.

## DISCUSSION

Regarding the treatment of infections related to CIED, all selected studies performed removal of the infected device, preferably by percutaneous extraction. Surgical removal was indicated in cases of failure of transvenous extraction, large vegetations, involvement of valves or lesions that developed during the percutaneous procedure^[16,20,25]^.

Percutaneous technique has been the preferred method of lead extraction according to the literature. Although surgical removal presented high mortality rates, it is important to consider that this type of procedure is associated with severe patients and complications related to infection or previous procedure, when compared to lower risk patients submitted to percutaneous lead extraction^[20-25]^.

Prior to the routine use of percutaneous extraction techniques, the infections of the devices were conservatively treated only with antibiotics. This strategy was associated with a very high mortality rate, forcing physicians to rethink treatment options^[23]^. Grammes et al.^[21]^ reported that in nine retrospective studies, the mortality rate was 41% for patients treated with antibiotics alone and 19% for patients treated with antibiotic therapy and device removal. Another study demonstrated that the extraction of the system was related to better survival after one year (19.9% and 38.2%, respectively, for the groups with and without extraction)^[28]^.

Le et al.^[24]^ demonstrated an increase in one-year mortality in the minority of patients whose infected device was not extracted because of the high risk of complications or low life expectancy and observed a three-fold increase in mortality when delayed extraction occurred. These data corroborate the current recommendations to extract the infected device, regardless of local or systemic clinical presentations^[10]^.

The main obstacles to extraction are tissue binding sites along the course of the lead and the interface between the lead tip and endocardium. In most cases, there is more than one binding site, and simple traction of the proximal lead is not transmitted to its distal tip. In these circumstances, there is a significant risk of rupture of the lead and fibrous tissue, along with all the complications that may result from rupture^[29]^.

With the adoption of new extraction techniques, success rates and safety procedures have notably improved^[21,30,31]^. In one such technique, a locking stylet inserts into the lumen of the lead and spreads traction forces along its body to the tip. Other traction devices include snares, sutures and grasping devices. However, it is often necessary to use these stylets together with sheaths to directly release the fibrous tissues^[29]^.

Wilkoff et al.^[30]^ performed a prospective randomized clinical trial with a sample of 301 patients (PLEXES trial) and verified that the use of a laser sheath was associated with a better success rate in extraction compared to the group that did not use this technique (94% and 64%, respectively). In addition, there was no significant increase in major complications. A further multicenter retrospective study, Lexicon, which also used laser sheath on lead extraction in a large number of patients (n=1,449), had a 96.5% removal success rate, and showed major adverse events in 1.4% of patients, with a mortality of 0.28% related to the procedure^[31]^. Both studies demonstrate the efficacy of the laser in device extraction, as well as the low rates of complications related to its use. In the studies analyzed in this review, simple manual traction was used as the main technique for removal of the device in percutaneous extraction, but other techniques, such as laser sheath, locking stylets and dilator sheaths, were required in some cases^[14-17,22,24,25]^.

In addition, the studies showed an average success rate of 92.4% in the complete percutaneous removal of infected devices^[12,13,16,18,22,24,25]^. At the same time, success rates of percutaneous extraction from 93% to 97% were reported in other large cohorts of patients^[32-34]^. Adequate device extraction is important, as inadequate device extraction is one of the main predisposing factors for the recurrence of infection^[20]^.

Percutaneous extraction is now accepted as a safe procedure, due to technical and surgical advances over the years^[35]^. The percutaneous extraction mortality rates reported in the literature ranged from 0.1% to 0.6%, and the rate of major complications ranged from 1.4% to 1.9%^[33,34,36,37]^. In this review, it was observed that the mortality associated with the CIED removal procedure ranged from 0.4% to 3.6%^[12,17,18,21,25-27]^ and that the mean incidence of major complications was 2.9%, which is a somewhat higher value than those described in the literature. Only the mortality rates obtained in Pichlmaier et al.^[25]^ and Knigina et al.^[17]^ were higher than the overall mortality rate.

In 191 patients, complications related to the extraction procedures were identified, of which 60 (31.4%) were major complications and 131 (68.6%) were minor complications. Le et al.^[24]^ observed that patients with complications were four times more likely to die when compared to those with a successful procedure.

The in-hospital mortality of patients with CIED infection ranged from 2% to 10.8% in this review, with an average of 5.4%. Grammes et al.^[21]^ presented 10% mortality in the first 30 days after device extraction, but this value did not reflect mortality directly related to the procedure since it occurred in a subgroup of critically ill patients with extensive comorbidities. Tarakji et al.^[26]^ showed an in-hospital mortality of 25 patients, and of these, only two (8%) patients died from causes related to device extraction, which corroborates the idea that postoperative mortality may not reflect procedure-related mortality.

Regarding the long-term mortality of these patients, some studies have demonstrated the following predictors: systemic embolization; moderate to severe tricuspid regurgitation; and comorbidities, such as chronic renal failure, dementia, advanced heart failure, presence of signs and symptoms of systemic infection, advanced age, use of anticoagulants, corticosteroids or immunosuppressive therapy^[13,19,24,26]^.

Despite the many advantages of percutaneous extraction, unfortunately this procedure presents higher costs when compared to surgical removal. Advanced techniques such as stylets and laser sheath have excessive costs and this is a major limitation of its use^[30,31]^.

### Limitations of the Study

The authors considered the lack of data standardization in the selected studies to be a limitation of this review because it is difficult to compare datasets. Some studies did not present data on procedure-related complications, long-term mortality, or differences in the results compared to a percutaneous technique with thoracotomy. Another limitation of was the lack of even comparative studies between the costs of percutaneous removal versus surgical removal.

## CONCLUSION

This systematic review revealed the importance of percutaneous extraction of infected cardiac electronic devices for adequate remission of infection. It also presented low rates of complications and mortality related to percutaneous removal.

**Table:** 

Authors' roles & responsibilities	
ASMJR	Substantial contributions to the conception or design of the work; or the acquisition, analysis, or interpretation of data for the work and agreement to be accountable for all aspects of the work in ensuring that questions related to the accuracy or integrity of any part of the work are appropriately investigated and resolved; final approval of the version to be published
TRM	Substantial contributions to the conception or design of the work; or the acquisition, analysis, or interpretation of data for the work; drafting the work or revising it critically for important intellectual content final approval of the version to be published
AOAM	Drafting the work or revising it critically for important intellectual content; agreement to be accountable for all aspects of the work in ensuring that questions related to the accuracy or integrity of any part of the work are appropriately investigated and resolved; final approval of the version to be published
